# Factors associated with job crafting among Chinese clinical nurses-a systematic review and meta-analysis

**DOI:** 10.3389/fpubh.2026.1961368

**Published:** 2026-10-19

**Authors:** Fei Guo, Wei Zhao, Mei Zhou, Wen-Nv Hao

**Affiliations:** 1 School of Nursing, Inner Mongolia Medical University, Hohhot, Inner Mongolia, China; 2 Department of Emergency, Affiliated Hospital of Inner Mongolia Medical University, Hohhot, Inner Mongolia, China

**Keywords:** associated factors, job crafting, meta-analysis, nurses, systematic review

## Abstract

**Objectives:**

To quantitatively evaluate factors associated with job crafting among nurses and to provide evidence for improving nurse well-being and developing targeted interventions.

**Methods:**

This review systematically searched PubMed, Web of Science, Embase, Cochrane Library, Scopus, CNKI, Wanfang, VIP, and China Biomedical Literature Database from the establishment of the databases to November 28, 2025. Two researchers independently screened literature and extracted data. The AHRQ cross-sectional study quality assessment tool was used for quality evaluation. The GRADE system was used to grade the certainty of evidence for each factor. RevMan 5.4 was used for meta-analysis.

**Results:**

A total of 16 cross-sectional studies were included, all conducted in China, involving 11,678 nurses. Meta-analysis showed that educational level (SMD = −0.30, 95% CI: −0.42 to −0.19, I^2^ = 29%), night shift frequency (SMD = 0.29, 95% CI: 0.18 to 0.40, I^2^ = 42%), and employment type (SMD = 0.56, 95% CI: 0.40 to 0.72, I^2^ = 0%) were significantly associated with job crafting. Self-efficacy (pooled *r* = 0.60, 95% CI: 0.33 to 0.79, I^2^ = 99%), professional identity (pooled *r* = 0.85, 95% CI: 0.62 to 0.95, I^2^ = 98%), perceived organizational support (pooled *r* = 0.57, 95% CI: 0.31 to 0.75, I^2^ = 97%), and empowering leadership (pooled *r* = 0.58, 95% CI 0.50 to 0.66, I^2^ = 93%) were positively correlated with job crafting. The pooled results for night shift work, age, professional title, and years of work experience were not statistically significant. However, each factor was based on only 2 to 4 studies, and some factors had extremely high heterogeneity (I^2^ = 93 to 99%) so results should be interpreted with caution.

**Conclusion:**

This study based on 16 Chinese cross-sectional studies preliminarily identified factors associated with job crafting among nurses. Educational level, night shift frequency, employment type, self-efficacy, professional identity, perceived organizational support, and empowering leadership were associated with job crafting. These findings may help nursing managers to focus on factors associated with job crafting and to explore intervention strategies. However, given the limited number of studies in each meta-analysis and the extremely high heterogeneity, the pooled estimates have substantial uncertainty, and the above associations should be considered exploratory. Future high-quality, longitudinal, or interventional studies are needed for verification.

**Systematic review registration:**

https://www.crd.york.ac.uk/prospero/display_record.php?ID=CRD420251270123.

## 1 Introduction

The global nursing shortage has been explicitly recognized by the World Health Organization and the International Council of Nurses as a global health emergency that poses a serious threat to the sustainability of health systems (1, 2). The World Health Organization, in its State of the World‘s Nursing 2025 report (2), noted that despite overall growth in the total number of nurses worldwide, uneven distribution of human resources, insufficient support for working conditions, and the impending wave of retirements all pose threats to the stability of the nursing workforce. Nurses are indispensable to the attainment of Universal Health Coverage, and as the ICN has explicitly stated, “there can be no health for all without nurses for all” (3). Yet the nursing workforce continues to be overworked, undervalued, and underprotected (4). Globally, the prevalence of burnout among nurses exceeds 50% (5), and turnover intention rates range from 30 to 50% (6, 7). These challenges not only compromise nurses’ individual physical and mental health and professional development, but also more directly threaten the quality of patient care and the equity and accessibility of public health services. Improving nurses’ occupational health and effectively sustaining workforce stability has therefore become an urgent priority that must be addressed in the field of global public health.

In this context, job crafting, as a proactive and bottom-up individual adaptive behavior, offers a promising new perspective for addressing the challenges outlined above. Job crafting refers to the process in which employees proactively redefine and adjust job tasks, cognitions, and relationship boundaries to better achieve a person-job fit (8). Research indicates that job crafting exerts a positive impact on the career development of nurses. At the individual level, job crafting significantly enhances nurses’ work engagement (9), innovative behavior (10), job satisfaction (11), and overall well-being (12). At the organizational level, it contributes to the optimization of nursing workflows, promotes team collaboration, and increases retention intention (13). Moreover, job crafting has been consistently demonstrated across multiple studies to directly influence quality of care and patient safety (14) and to reduce the occurrence of missed nursing care (15). Particularly in high-risk contexts such as infectious disease prevention and control and public health emergency responses, nurses with greater job crafting capacity exhibit stronger job embeddedness and better psychological adaptability (16). Thus, job crafting is not merely a protective factor for individual nurses’ career development; it is also a critical entry point for safeguarding the quality and safety of public health services from the perspective of nursing workforce strengthening. A thorough exploration and active promotion of job crafting among nurses therefore carries significant theoretical and practical value.

Currently, both domestic and international scholars have begun to pay attention to research on job crafting among clinical nurses and have preliminarily explored its associated factors. However, due to differences in study designs, measurement instruments, and sample characteristics, the associated factors examined across these studies remain fragmented and have not yet yielded consistent conclusions. Meta-analysis, as a high-level evidence synthesis method in evidence-based medicine, can overcome the limitations of individual studies and resolve inconsistent findings (17). It can thereby provide a more reliable evidence base for health policymakers. To our knowledge, no systematic review or meta-analysis to date has quantitatively synthesized the factors associated job crafting among clinical nurses. Therefore, this study used a systematic review and meta-analysis to quantitatively synthesize factors associated with job crafting among clinical nurses. The findings aim to provide exploratory references for nursing management practice and future intervention research.

## 2 Materials and methods

### 2.1 Research design and registration

This study strictly adheres to the Preferred Reporting Items for Systematic Reviews and Meta-Analyses (PRISMA 2020) statement (see Supplementary Table S1). The research protocol has been registered in PROSPERO (Registration number: CRD420251270123). The review question was framed using the PICOS approach: Population (clinical nurses), Phenomenon of Interest (factors associated with job crafting), Comparator (not applicable for correlational analyses; group comparisons for categorical variables), Outcomes (job crafting), and Study design (cross-sectional studies).

### 2.2 Literature search strategy

We systematically searched nine databases, including PubMed, Cochrane Library, Web of Science, Embase, Scopus, China National Knowledge Infrastructure (CNKI), Wanfang Data, VIP, and China Biomedical Literature Database (CBM). The search period was from database inception to November 28, 2025. Search terms included nurses, job crafting, and related terms. The search strategy combined subject headings and free-text terms and was adapted to each database. For example, the search strategy for PubMed is shown in Figure 1. The complete search strategy is provided in Supplementary Table S2.

**Figure 1:**
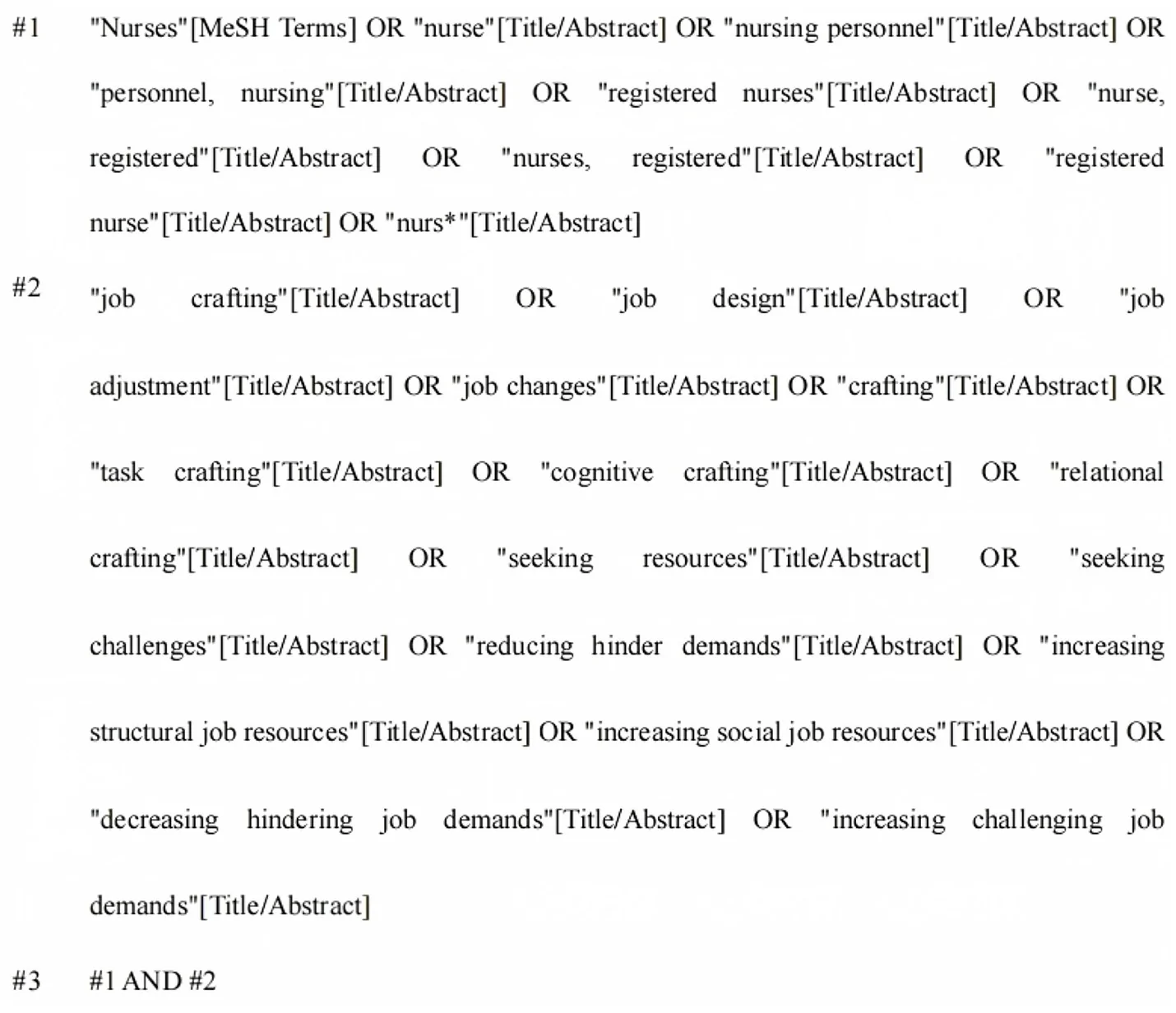
Search strategy for PubMed.

### 2.3 Inclusion and exclusion criteria

Inclusion criteria: (1) Study design: cross-sectional studies; (2) Participants: clinical nurses; (3) Outcomes: factors associated with job crafting among nurses were reported; (4) Language: Chinese or English; (5) Measurement tool: job crafting was assessed using a specific job crafting scale.

Exclusion criteria: (1) Duplicate publications; (2) Studies with unavailable full text or insufficient data; (3) Studies that did not report factors associated with job crafting among nurses; (4) Reviews, commentaries, conference abstracts, and dissertations.

### 2.4 Literature screening and data extraction

Two investigators independently performed the literature screening and data extraction. Initially, duplicate studies were excluded using NoteExpress software. Subsequently, preliminary screening was conducted by reviewing titles and abstracts, followed by a full-text review for further evaluation. Disagreements were resolved through consultation with a third investigator. Extracted data encompassed key information from the included studies: first author, publication year, sample size, study region, study design, assessment tools, and associated factors.

### 2.5 Quality assessment of the included studies

The Agency for Healthcare Research and Quality (AHRQ) cross-sectional study quality assessment criteria were used to evaluate the overall methodological quality of the included studies (18). This tool contains 11 items. Each item was rated as yes, no, or unclear and scored 1, 0, and 0, respectively. The total score was the sum of all item scores. Studies were classified into three quality levels, namely high quality with scores of 8 to 11, moderate quality with scores of 4 to 7, and low quality with scores of 0 to 3 (19). Two researchers independently assessed study quality. Disagreements were resolved through discussion or consultation with a third researcher until consensus was reached.

### 2.6 GRADE assessment of evidence certainty

The Grading of Recommendations Assessment, Development and Evaluation system (GRADE) was used to assess the certainty of evidence for each factor included in the meta-analysis (20). The initial certainty level for observational cross-sectional designs was low. Domains for downgrading included risk of bias, inconsistency, indirectness, imprecision, and publication bias. Domains for upgrading included large effect size, dose–response relationship, and residual confounding that may reduce the effect (21). Two researchers independently assessed the evidence. Disagreements were resolved through discussion or by a third researcher. The final certainty of evidence was classified as high, moderate, low, or very low.

### 2.7 Statistical analysis

RevMan 5.4 was used for statistical analysis. For continuous variables, standardized mean difference (SMD) and its 95% confidence interval (CI) were used as the pooled effect size. A positive SMD indicated a higher score in the comparison group than in the reference group, whereas a negative SMD indicated a lower score. For correlational variables, unadjusted bivariate correlation coefficients (Pearson or Spearman r) reported in each study were extracted, pooled after Fisher’s Z transformation, and then back-transformed to pooled r and 95% CIs. Heterogeneity among studies was assessed using the I^2^ statistic. If *p* > 0.1 and I^2^ ≤ 50%, heterogeneity was considered acceptable, and a fixed-effects model was used. If *p* ≤ 0.1 or I^2^ > 50%, significant heterogeneity was indicated, and a random-effects model was used. Leave-one-out sensitivity analyses were performed for pooled results with statistical significance, using the same effect model as the primary analysis. When a factor included ≥ 10 studies, Egger’s test was used to assess publication bias, with a significance level of α = 0.05 (22).

Meta-analysis was performed for the same factor reported in two or more studies (23). All included studies were cross-sectional studies of Chinese clinical nurses, with consistent study participants, design, and outcome measures. Although the job crafting scale included two versions developed by Tims et al. (24) and Dvorak (25), respectively, and the measurement tools for perceived organizational support and empowering leadership also differed across studies, all assessed the same construct. Therefore, despite the high I^2^ for some factors, the studies were considered sufficiently comparable in clinical and methodological terms to warrant exploratory pooling (26). Pooled estimates were used to summarize the overall trend of existing evidence, and the results should be interpreted as exploratory only. For factors reported in only one study, a narrative synthesis was performed.

## 3 Results

### 3.1 Literature screening process and results

A total of 2,070 potentially relevant articles were initially identified. After removing duplicates using NoteExpress and manual screening, 1,442 records remained. By reading the titles and abstracts, 1,222 documents that did not meet the requirements were excluded, leaving 220 documents for which the full-text was retrieved for detailed evaluation. Ultimately, 16 studies were included in this systematic review and meta-analysis. The literature screening process is detailed in Figure 2.

**Figure 2:**
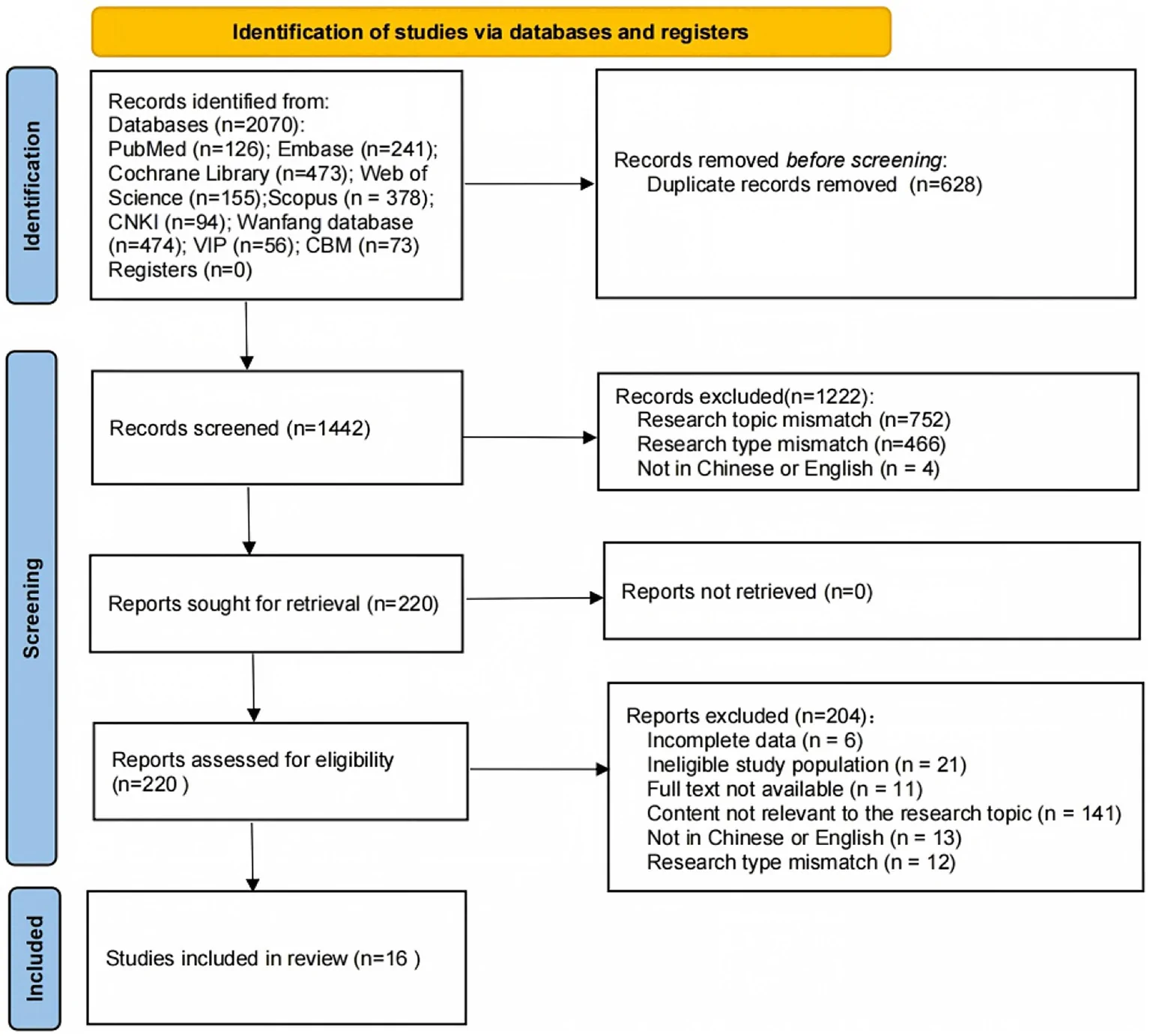
PRISMA 2020 flow diagram for literature screening.

### 3.2 Basic characteristics of the included studies

A total of 16 cross-sectional studies were included, with a total sample size of 11,678 nurses. All studies were conducted in different provinces of China. The publication years ranged from 2020 to 2025, and sample sizes ranged from 163 to 3,525. Among them, 9 studies used the four-dimensional Job Crafting Scale developed by Tims et al. (24), and the remaining 7 studies used the three-dimensional Job Crafting Scale developed by Dvorak (25). Both scales contain 21 items and use a 5-point Likert scale, with total scores ranging from 21 to 105; higher scores indicate higher levels of job crafting. The basic characteristics of the included studies are summarized in Table 1.

**Table 1:** Basic characteristics of included studies.

Author, year	Sample size	Study region	Design	Instrument	Associated factors
Chen L. L. et al., 2023 (42)	414	Zhejiang, China	Cross-sectional	Tims’ Job Crafting Scale	①, ②, ③
Wang B. J. et al., 2025 (36)	356	Tianjin / Shandong, China	Cross-sectional	Tims’ Job Crafting Scale	④, ⑤, ⑥, ⑦
Lian Q. et al., 2025 (35)	210	Guangdong, China	Cross-sectional	Tims’ Job Crafting Scale	④, ⑧, ⑨, ⑩
Duan H. L. et al., 2024 (40)	163	Henan, China	Cross-sectional	Tims’ Job Crafting Scale	⑪, ⑫, ⑬, ⑭
Duan S. Y. et al., 2024 (30)	431	Xinjiang, China	Cross-sectional	Tims’ Job Crafting Scale	④, ⑧, ⑮, ⑯, ⑰
Fang H. L. et al., 2021 (27)	231	Henan, China	Cross-sectional	Tims’ Job Crafting Scale.	⑮, ⑱, ⑲, ⑳
Jiang F. R. et al., 2023 (37)	509	Hunan, China	Cross-sectional	Tims’ Job Crafting Scale	⑧, ㉑, ⑳
Li M. L. et al., 2022 (31)	884	Hubei / Jiangxi, China	Cross-sectional	Dvorak’s Job Crafting Scale	⑳,㉒, ㉓, ㉔
Li G. Z. et al., 2024 (38)	1,527	Shandong, China	Cross-sectional	Tims’ Job Crafting Scale	⑳, ㉕, ㉖
Luo Z. J. 2023 (33)	206	Hubei, China	Cross-sectional	Dvorak’s Job Crafting Scale	⑨, ㉓, ㉗, ㉘
Tian Q. 2020 (41)	216	Shandong, China	Cross-sectional	Tims’ Job Crafting Scale	㉙, ㉚
Wu M. R. et al., 2025 (28)	1,328	Sichuan, China	Cross-sectional	Dvorak’s Job Crafting Scale	⑮, ㉛, ㉜, ㉝
Xu X. X. et al., 2023 (39)	3,525	Xinjiang, China	Cross-sectional	Dvorak’s Job Crafting Scale	㉛, ㉞, ㉟, ㊱, ㊲
Yang Y. et al., 2024 (32)	562	Sichuan / Chongqing, China	Cross-sectional	Dvorak’s Job Crafting Scale	㉓, ㊳, ㊴, ㊵, ㊶, ㊷
Zhao X. et al., 2025 (29)	641	Shaanxi, China	Cross-sectional	Dvorak’s Job Crafting Scale	⑮, ⑱, ㉓, ㊳, ㉝, ㊸
Zhong Y. et al., 2025 (34)	475	Hunan, China	Cross-sectional	Dvorak’s Job Crafting Scale	⑨, ㉗, ㊳, ㊹

① Career aspiration; ② Organizational climate; ③ Job position; ④ Employment type; ⑤ One-way commuting time; ⑥ Workplace social capital; ⑦ Person-organization fit; ⑧ Perceived organizational support; ⑨ Professional identity; ⑩ Hospital grade; ⑪ Number of children; ⑫ Dedication; ⑬ Competence; ⑭ Autonomy; ⑮ Educational level; ⑯ Self-rated job satisfaction; ⑰ Proactive personality; ⑱ Age; ⑲ Monthly income; ⑳ Self-efficacy; ㉑ Intrinsic motivation; ㉒ Marital status; ㉓ Professional title; ㉔ Character strengths; ㉕ Perceived social support; ㉖ Gender role conflict; ㉗ Night shift work (yes/no); ㉘ Work engagement; ㉙ Structural empowerment; ㉚ Psychological empowerment; ㉛ Empowering leadership; ㉜ Nurse manager humanistic care behavior; ㉝ Frequency of night shifts; ㉞ Ethnicity; ㉟ Initial employment motivation; ㊱ Regulatory focus; ㊲ Job performance; ㊳ Years of work experience; ㊴ Leisure crafting; ㊵ Psychological capital; ㊶ Decent work perception; ㊷ Psychological detachment; ㊸ Self-perception of department continuing education; ㊹ Empowerment.

### 3.3 Quality assessment

All 16 included studies were of moderate quality (total score 5–7), with no high or low quality studies, and all were included. At the item level, the included studies reported sample source, inclusion and exclusion criteria, and data collection period adequately. However, reporting on participant continuity, assessor blinding, and missing data handling was insufficient, which may introduce selection bias and information bias. In addition, although all studies used multivariable regression to control for confounders, the cross-sectional design may render outcome variables susceptible to confounding. In summary, these biases and residual confounding may affect the accuracy of pooled estimates. The quality assessment results are shown in Table 2.

**Table 2:** Results of the AHRQ item-level quality assessment for the included studies.

Study	Item 1	Item 2	Item 3	Item 4	Item 5	Item 6	Item 7	Item 8	Item 9	Item 10	Item 11	Total	Quality
Chen L. L. et al., 2023 (42)	Y	Y	Y	N	U	Y	Y	Y	U	Y	NA	7	Moderate
Wang B. J. et al., 2025 (36)	Y	Y	Y	N	U	Y	Y	Y	U	Y	NA	7	Moderate
Lian Q. et al., 2025 (35)	Y	Y	Y	N	U	Y	Y	Y	U	Y	NA	7	Moderate
Duan H. L. et al., 2024 (40)	Y	Y	Y	N	U	Y	Y	Y	U	Y	NA	7	Moderate
Duan S. Y. et al., 2024 (30)	Y	Y	Y	N	U	Y	Y	Y	U	Y	NA	7	Moderate
Fang H. L. et al., 2021 (27)	Y	Y	Y	N	U	Y	Y	Y	U	Y	NA	7	Moderate
Jiang F. R. et al., 2023 (37)	Y	Y	Y	N	U	Y	Y	Y	U	Y	NA	7	Moderate
Li M. L. et al., 2022 (31)	Y	Y	Y	N	U	Y	Y	Y	U	Y	NA	7	Moderate
Li G. Z. et al., 2024 (38)	Y	Y	Y	N	U	Y	Y	Y	U	N	NA	6	Moderate
Luo Z. J. 2023 (33)	Y	Y	Y	N	U	Y	Y	Y	U	Y	NA	7	Moderate
Tian Q. 2020 (41)	Y	Y	Y	N	U	U	Y	Y	U	Y	NA	6	Moderate
Wu M. R. et al., 2025 (28)	Y	Y	Y	N	U	Y	Y	Y	U	Y	NA	7	Moderate
Xu X. X. et al., 2023 (39)	Y	Y	Y	N	U	Y	Y	Y	U	Y	NA	7	Moderate
Yang Y. et al., 2024 (32)	Y	Y	Y	N	U	Y	Y	Y	U	Y	NA	7	Moderate
Zhao X. et al., 2025 (29)	Y	Y	Y	N	U	Y	Y	Y	U	Y	NA	7	Moderate
Zhong Y. et al., 2025 (34)	Y	Y	Y	N	U	Y	Y	Y	U	N	NA	6	Moderate

Y, yes; N, no; U, unclear; NA, not applicable. Items 1–11: (1) source of information; (2) inclusion and exclusion criteria; (3) time period for identifying patients; (4) consecutive subjects; (5) masking of evaluators; (6) quality assurance assessments; (7) reasons for patient exclusions; (8) confounding control; (9) missing data handling; (10) response rate and data completeness; (11) follow-up and incomplete data.

### 3.4 Meta-analysis results

A total of 11 associated factors were extracted and pooled separately. For clarity, these factors were grouped into three categories: occupational characteristics, psychological factors, and organizational factors. The pooled results for each factor are summarized in Table 3.

**Table 3:** Meta-analysis results of influencing factors of job crafting among nurses.

Influencing factor	Group	Number of studies	Heterogeneity test		Effect model	Meta-analysis results			
			I^2^(%)	*p* value		Pooled effect size (95% CI)	Pooled r (95% CI)	*p* value	Z value
Education level	junior college degree or below / bachelor’s degree or above	4	29	0.24	FE	SMD = −0.30 [−0.42, −0.19]		5.30	<0.00001
Professional titles	nurse practitioner and below / senior nurse practitioner and above	4	96	<0.00001	RE	SMD = −0.48 [−1.03, 0.07]		1.72	0.09
Years of work experience	≤10 years / >10 years	3	95	<0.00001	RE	SMD = −0.16 [−0.65, 0.32]		0.66	0.51
Night-shift frequencies	≤6 times / >6 times	2	42	0.19	FE	SMD = 0.29 [0.18, 0.40]		5.25	<0.00001
Employment types	permanent / non-permanent	3	0	0.52	FE	SMD = 0.56 [0.40, 0.72]		6.92	<0.00001
Self-efficacy	-	3	99	<0.00001	RE	Fisher’ Z = 0.70 [0.34, 1.06]	Pooled *r* = 0.60 [0.33, 0.79]	3.82	0.0001
Professional identity	-	3	98	<0.00001	RE	Fisher’ Z = 1.27 [0.72, 1.82]	Pooled *r* = 0.85 [0.62, 0.95]	4.53	<0.00001
Perceived organizational support	-	3	97	<0.00001	RE	Fisher’ Z = 0.65 [0.32, 0.97]	Pooled *r* = 0.57 [0.31, 0.75]	3.88	0.0001
Empowering leadership	-	2	93	<0.00001	RE	Fisher’ Z = 0.67 [0.55, 0.79]	Pooled *r* = 0.58 [0.50, 0.66]	10.92	<0.00001

FE, fixed-effect model; RE, random-effects model.

#### 3.4.1 Occupational characteristics factors

Educational level. Four studies (27–30) reported the relationship between educational level and job crafting. Participants were divided into two groups: junior college or below (comparison group) and bachelor’s degree or above (reference group). The heterogeneity test showed I^2^ = 29% (*p* = 0.24), indicating acceptable heterogeneity, and a fixed-effects model was used. Meta-analysis showed that nurses with junior college or below had lower job crafting scores than those with bachelor’s degree or above, with a statistically significant difference (SMD = −0.30, 95% CI: −0.42 to −0.19, Z = 5.30, *p* < 0.00001), as shown in Figure 3.

**Figure 3:**
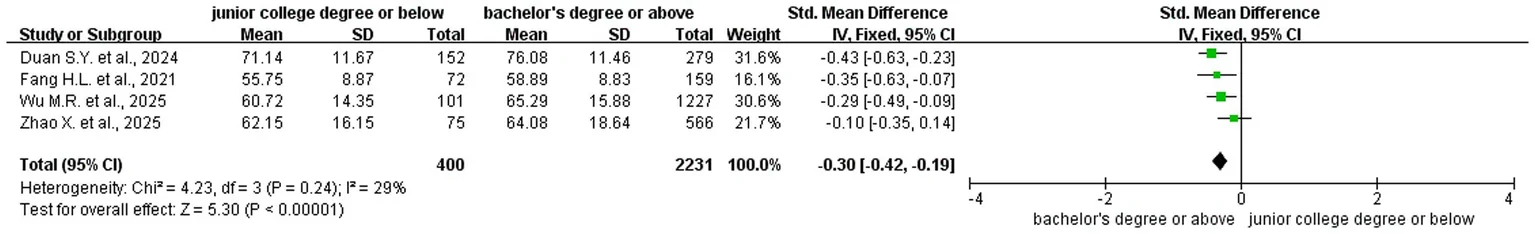
Forest plot of educational level and job crafting.

Age. Two studies (27, 29) reported the relationship between age and job crafting. Participants were divided into two groups: <30 years (comparison group) and ≥30 years (reference group). The heterogeneity test showed I^2^ = 97% (*p* < 0.00001), indicating significant heterogeneity, and a random-effects model was used. Meta-analysis showed no statistically significant difference in job crafting scores among nurses of different ages (SMD = −0.22, 95% CI: −1.11 to 0.68, Z = 0.47, *p* = 0.64), as shown in Figure 4.

**Figure 4:**
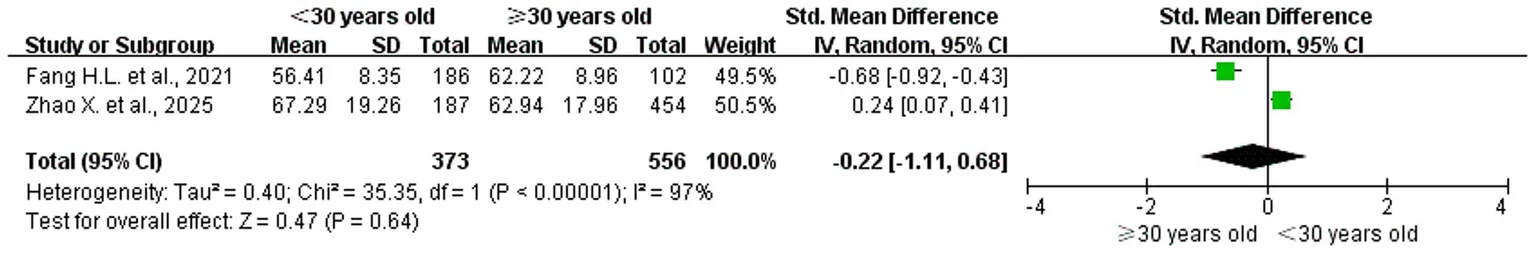
Forest plot of age and job crafting.

Professional title. Four studies (29, 31–33) reported the relationship between professional title and job crafting. Participants were divided into two groups: senior nurse and below (comparison group) and supervisor nurse and above (reference group). The heterogeneity test showed I^2^ = 96% (*p* < 0.00001), indicating significant heterogeneity, and a random-effects model was used. Meta-analysis showed no statistically significant difference in job crafting scores among nurses with different professional titles (SMD = −0.48, 95% CI: −1.03 to 0.07, Z = 1.72, *p* = 0.09), as shown in Figure 5.

**Figure 5:**
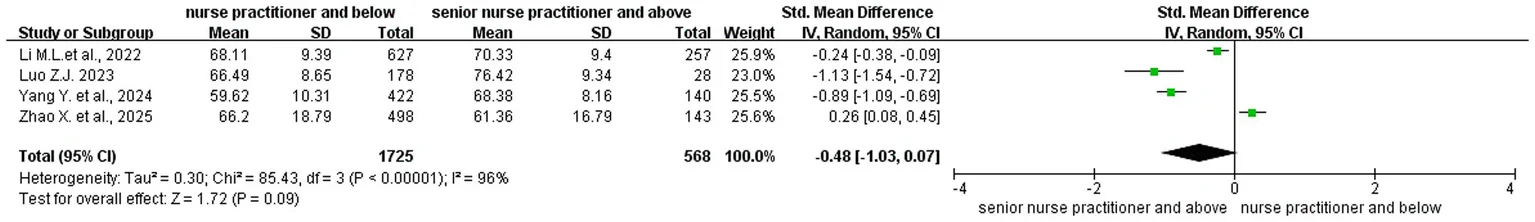
Forest plot of professional title and job crafting.

Night shift status. Two studies (33, 34) reported the relationship between night shift status and job crafting. Participants were divided into two groups: working night shifts (comparison group) and not working night shifts (reference group). The heterogeneity test showed I^2^ = 97% (*p* < 0.00001), indicating significant heterogeneity, and a random-effects model was used. Meta-analysis showed no statistically significant association between night shift status and job crafting among nurses (SMD = −0.97, 95% CI: −2.01 to 0.07, Z = 1.82, *p* = 0.07), as shown in Figure 6.

**Figure 6:**
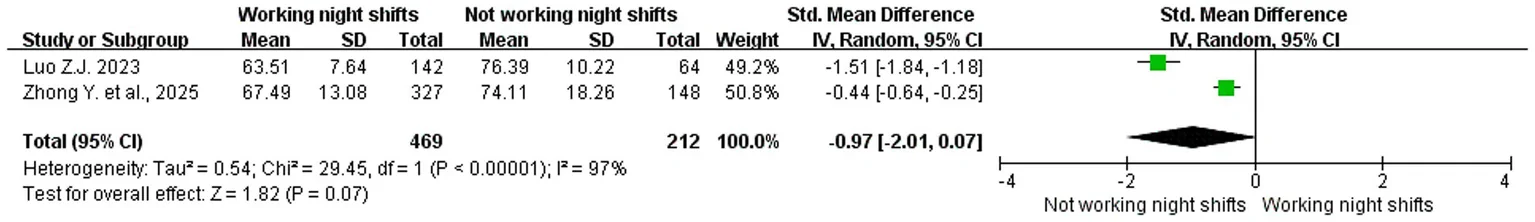
Forest plot of night shift status and job crafting.

Years of work experience. Three studies (29, 32, 34) reported the relationship between years of work experience and job crafting. Participants were divided into two groups: ≤10 years (comparison group) and >10 years (reference group). The heterogeneity test showed I^2^ = 95% (*p* < 0.00001), indicating significant heterogeneity, and a random-effects model was used. Meta-analysis showed no statistically significant difference in job crafting scores among nurses with different years of work experience (SMD = −0.16, 95% CI: −0.65 to 0.32, Z = 0.66, *p* = 0.51), as shown in Figure 7.

**Figure 7:**
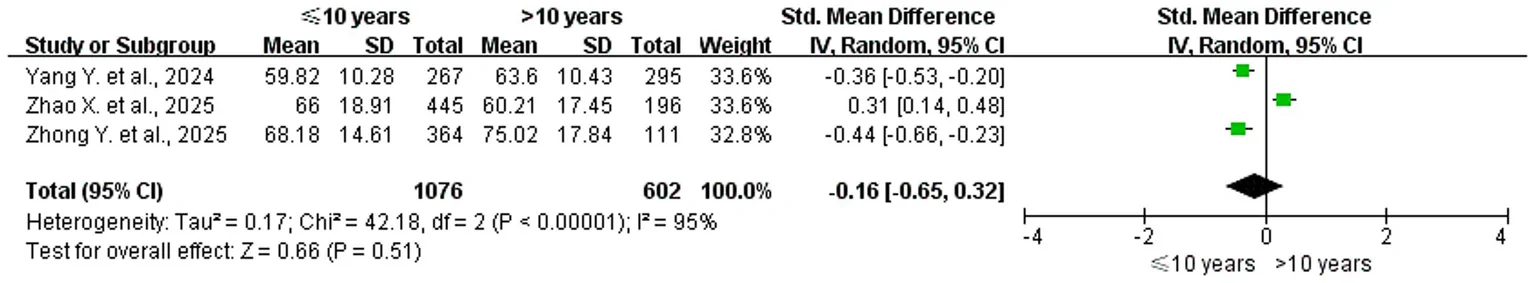
Forest plot of years of work experience and job crafting.

Night shift frequency. Two studies (28, 29) reported the relationship between night shift frequency and job crafting. Participants were divided into two groups: ≤6 night shifts per month (comparison group) and >6 night shifts per month (reference group). The heterogeneity test showed I^2^ = 42% (*p* = 0.19), indicating acceptable heterogeneity, and a fixed-effects model was used. Meta-analysis showed that nurses with ≤6 night shifts per month had higher job crafting scores than those with >6 night shifts per month, with a statistically significant difference (SMD = 0.29, 95% CI: 0.18 to 0.40, Z = 5.25, *p* < 0.00001), as shown in Figure 8.

**Figure 8:**
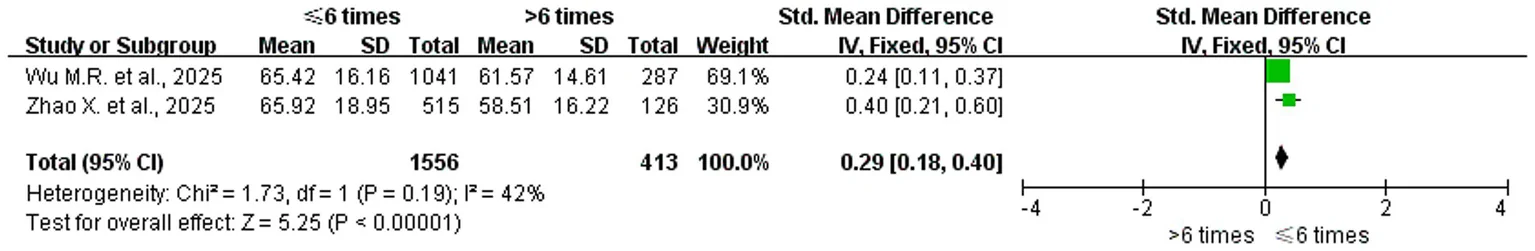
Forest plot of night shift frequency and job crafting.

Employment type. Three studies (30, 35, 36) reported the relationship between employment type and job crafting. Participants were divided into two groups: permanent staff (comparison group) and non-permanent staff (reference group). The heterogeneity test showed I^2^ = 0% (*p* = 0.52), indicating acceptable heterogeneity, and a fixed-effects model was used. Meta-analysis showed that permanent nurses had higher job crafting scores than non-permanent nurses, with a statistically significant difference (SMD = 0.56, 95% CI: 0.40 to 0.72, Z = 6.92, *p* < 0.0001), as shown in Figure 9.

**Figure 9:**
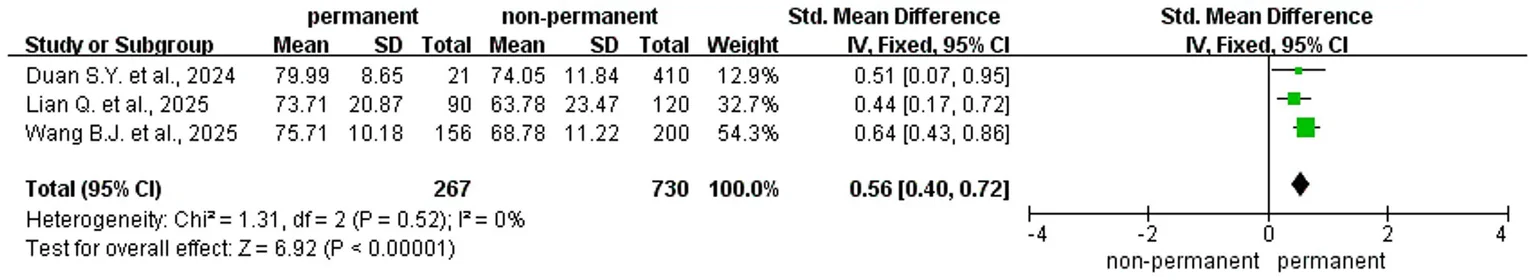
Forest plot of employment type and job crafting.

#### 3.4.2 Psychological factors

Self-efficacy. Three studies (31, 37, 38) examined the correlation between self-efficacy and job crafting. Heterogeneity testing showed I^2^ = 99% (*p* < 0.00001), indicating significant heterogeneity, and a random-effects model was used. After Fisher’s Z transformation, pooled analysis showed a positive correlation between self-efficacy and job crafting, with a statistically significant association (Fisher’s Z = 0.70, 95% CI: 0.34 to 1.06, Z = 3.82, *p* = 0.0001). After back-transformation, the pooled r was 0.60 (95% CI: 0.33 to 0.79), as shown in Figure 10.

**Figure 10:**
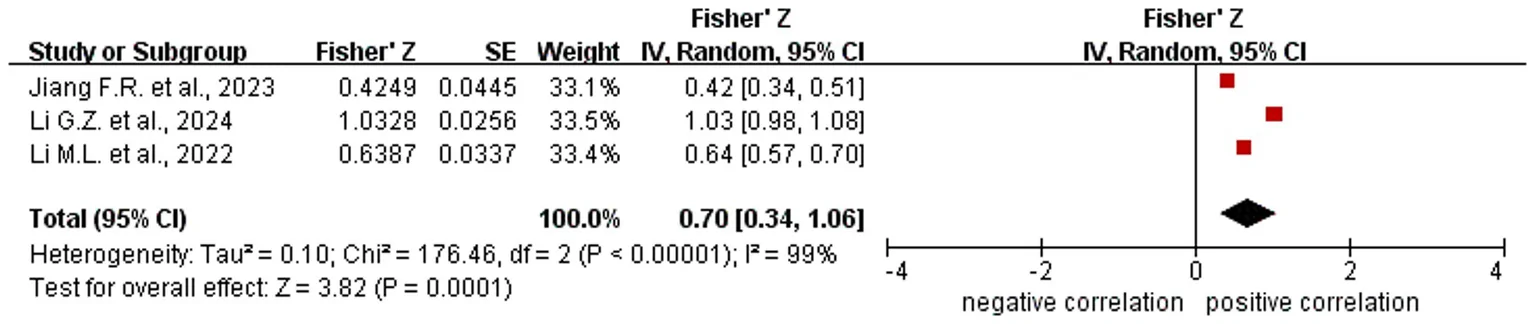
Forest plot of self-efficacy and job crafting.

Professional identity. Three studies (33–35) examined the correlation between professional identity and job crafting. Heterogeneity testing showed I^2^ = 98% (*p* < 0.00001), indicating significant heterogeneity, and a random-effects model was used. After Fisher’s Z transformation, pooled analysis showed a positive correlation between professional identity and job crafting, with a statistically significant association (Fisher’s Z = 1.27, 95% CI: 0.72 to 1.82, Z = 4.53, *p* < 0.00001). After back-transformation, the pooled r was 0.85 (95% CI: 0.62 to 0.95), as shown in Figure 11.

**Figure 11:**
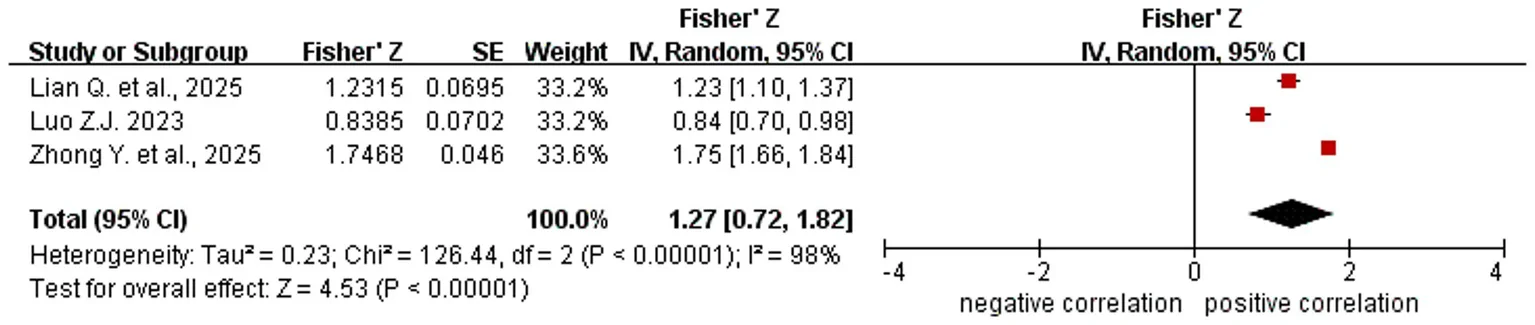
Forest plot of professional identity and job crafting.

#### 3.4.3 Organizational factors

Perceived organizational support. Three studies (30, 35, 37) examined the correlation between perceived organizational support and job crafting. Heterogeneity testing showed significant heterogeneity (I^2^ = 97%, *p* < 0.00001), and a random-effects model was used. After Fisher’s Z transformation, pooled analysis showed a positive correlation between perceived organizational support and job crafting, with a statistically significant association (Fisher’s Z = 0.65, 95% CI: 0.32 to 0.97, Z = 3.88, *p* = 0.0001). After back-transformation, the pooled r was 0.57 (95% CI: 0.31 to 0.75), as shown in Figure 12.

**Figure 12:**
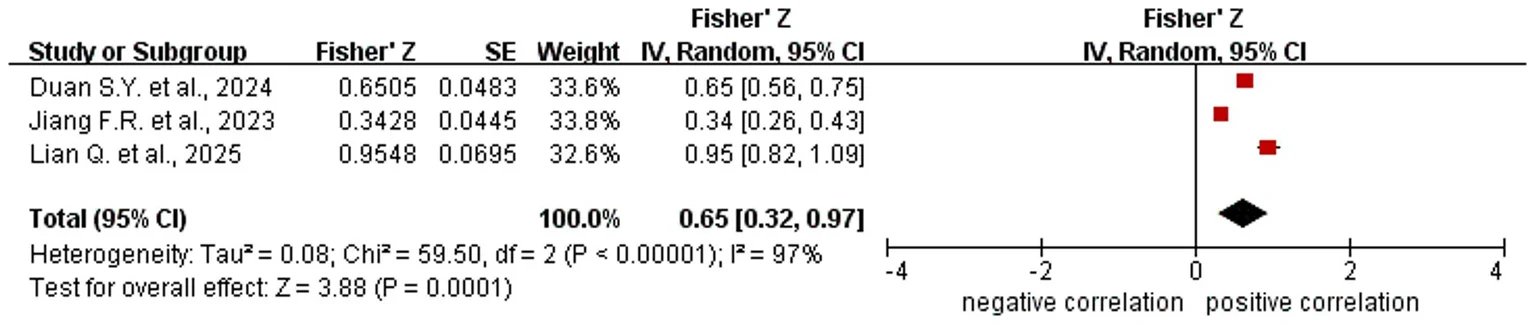
Forest plot of perceived organizational support and job crafting.

Empowering leadership. Two studies (28, 39) examined the correlation between empowering leadership behavior and job crafting. Heterogeneity testing showed significant heterogeneity (I^2^ = 93%, *p* = 0.00001), and a random-effects model was used. After Fisher’s Z transformation, pooled analysis showed a positive correlation between empowering leadership and job crafting, with a statistically significant association (Fisher’s Z = 0.67, 95% CI: 0.55 to 0.79, Z = 10.92, *p* < 0.00001). After back-transformation, the pooled r was 0.58 (95% CI: 0.50 to 0.66), as shown in Figure 13.

**Figure 13:**
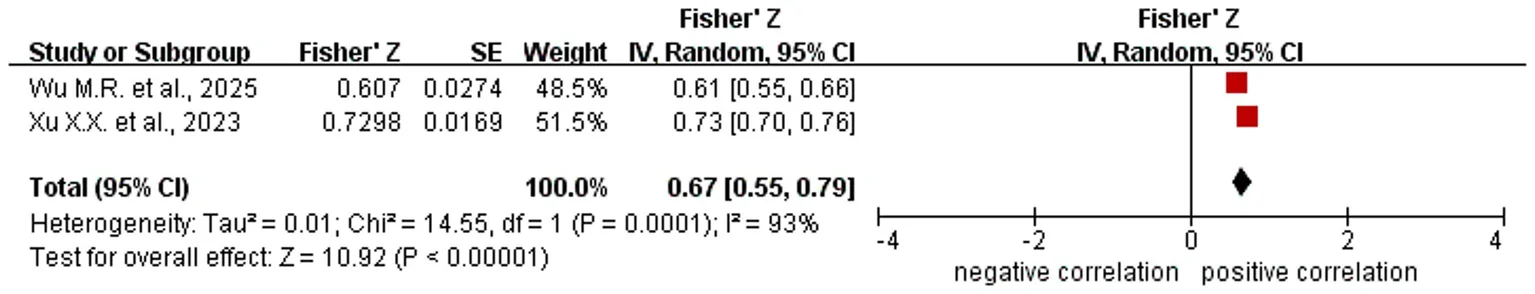
Forest plot of empowering leadership and job crafting.

### 3.5 Descriptive analysis

Factors reported in only a single study were summarized narratively. Fang et al. (27) measured self-efficacy but did not report its correlation with job crafting; therefore, this study was not included in the pooled analysis. Zhong et al. (34) reported a positive correlation between psychological empowerment and job crafting (*r* = 0.889); however, psychological empowerment was not pooled because it differs conceptually from empowering leadership. In addition, Duan et al. (40) reported that the number of children, dedication, competence, and autonomy were associated with job crafting among nurses. Tian (41) reported that structural empowerment and psychological empowerment were associated with job crafting. Chen et al. (42) reported that career aspiration, organizational climate, and position were also associated with job crafting.

### 3.6 GRADE evidence quality assessment

The certainty of evidence for the 7 factors significantly associated with job crafting in the meta-analysis was assessed using the GRADE system. All included studies were cross-sectional, and the initial certainty of evidence was rated as low. Due to limitations in the overall methodological quality of the included studies, the risk of bias domain was downgraded by 1 level for all factors. For self-efficacy, professional identity, perceived organizational support, and empowering leadership, significant heterogeneity was observed among studies (I^2^ = 93 to 99%), and the inconsistency domain was downgraded by 1 level. For employment type, self-efficacy, and perceived organizational support, the imprecision domain was downgraded by 1 level due to the small total sample size included in the analysis or wide confidence intervals. As each factor included fewer than 10 studies, publication bias could not be assessed. No upgrading factors were identified. Ultimately, the certainty of evidence for all 7 associated factors was rated as very low. The results of the GRADE assessment are presented in Table 4.

**Table 4:** GRADE assessment of the certainty of evidence.

Factors	No. of included studies	Study design	Initial certainty	Risk of bias	Inconsistency	Indirectness	Imprecision	Publication bias	Upgrading factors	Final certainty of evidence
Educational level	4	Cross-sectional	Low	Downgraded 1 level	Not downgraded	Not downgraded	Not downgraded	Unable to assess	None identified	Very low
Night shift frequency	2	Cross-sectional	Low	Downgraded 1 level	Not downgraded	Not downgraded	Not downgraded	Unable to assess	None identified	Very lowi
Employment type	3	Cross-sectional	Low	Downgraded 1 level	Not downgraded	Not downgraded	Downgraded 1 level	Unable to assess	None identified	Very low
Self-efficacy	3	Cross-sectional	Low	Downgraded 1 level	Downgraded 1 level	Not downgraded	Downgraded 1 level	Unable to assess	None identified	Very low
Professional identity	3	Cross-sectional	Low	Downgraded 1 level	Downgraded 1 level	Not downgraded	Not downgraded	Unable to assess	None identified	Very low
Perceived organizational support	3	Cross-sectional	Low	Downgraded 1 level	Downgraded 1 level	Not downgraded	Downgraded 1 level	Unable to assess	None identified	Very low
Empowering leadership	2	Cross-sectional	Low	Downgraded 1 level	Downgraded 1 level	Not downgraded	Not downgraded	Unable to assess	None identified	Very low

### 3.7 Sensitivity analysis and publication bias

Leave-one-out sensitivity analyses were performed for pooled results with statistical significance. The results showed that after sequentially excluding each study, the direction of the pooled effect did not reverse for educational level (SMD: −0.36 to −0.25), employment type (SMD: 0.46 to 0.62), perceived organizational support (Fisher’s Z: 0.50 to 0.80), self-efficacy (Fisher’s Z: 0.53 to 0.84), and professional identity (Fisher’s Z: 1.04 to 1.49), with limited changes in magnitude, suggesting that the pooled estimates were not driven by any single study. For empowering leadership and night shift frequency, only two studies were included; therefore, pooling was not possible after excluding either study. Detailed results are shown in Table 5. Because fewer than 10 studies were included for each factor, publication bias was not assessed separately for each factor.

**Table 5:** Results of leave-one-out sensitivity analyses.

Factor	Model used	Scenario	Study removed	k	Fisher’s Z (95% CI)	I^2^ (%)	τ^2^
Educational level	Fixed-effect (M-H)	Original (all studies)	—	4	SMD = −0.30 [−0.42, −0.19]	29.0	—
		Leave-one-out	Duan S. Y. et al., 2024 (30)	3	SMD = −0.25 [−0.38, −0.11]	3	—
		Leave-one-out	Fang H. L. et al., 2021 (27)	3	SMD = −0.29 [−0.42, −0.17]	51	—
		Leave-one-out	Wu M. R. et al., 2025 (28)	3	SMD = −0.31 [−0.44, −0.17]	52	—
		Leave-one-out	Zhao X. et al., 2025 (29)	3	SMD = −0.36 [−0.49, −0.23]	0	—
Night-shift frequency	Random-effects (DL)	Original (all studies)	—	2	SMD = 0.29 [0.18, 0.40]	42.0	—
		Leave-one-out	Wu M. R. et al., 2025 (28)	1	—	—	—
		Leave-one-out	Zhao X. et al., 2025 (29)	1	—	—	—
Employment type	Fixed-effect (M-H)	Original (all studies)	—	3	SMD = 0.56 [0.40, 0.72]	0.0	—
		Leave-one-out	Wang B. J. et al., 2025 (36)	2	SMD = 0.46 [0.23, 0.69]	0.0	—
		Leave-one-out	Lian Q. et al., 2025 (35)	2	SMD = 0.62 [0.42, 0.81]	0.0	—
		Leave-one-out	Duan S. Y. et al., 2024 (30)	2	SMD = 0.57 [0.40, 0.74]	20	—
Self-efficacy	Random-effects (DL)	Original (all studies)	—	3	Fisher’s Z = 0.70 [0.34, 1.06]	99	0.10
		Leave-one-out	Jiang F. R. et al., 2023 (37)	2	Fisher’s Z = 0.84 [0.45, 1.22]	99	0.08
		Leave-one-out	Li G. Z. et al., 2024 (38)	2	Fisher’s Z = 0.53 [0.32, 0.74]	93	0.02
		Leave-one-out	Li M. L. et al., 2022 (31)	2	Fisher’s Z = 0.73 [0.13, 1.33]	99	0.18
Professional identity	Random-effects (DL)	Original (all studies)	—	3	Fisher’s Z = 1.27 [0.72, 1.82]	98	0.23
		Leave-one-out	Zhong Y. et al., 2025 (34)	2	Fisher’s Z = 1.04 [0.65, 1.42]	94	0.07
		Leave-one-out	Luo Z. J. 2023 (33)	2	Fisher’s Z = 1.49 [0.99, 2.00]	97	0.13
		Leave-one-out	Lian Q. et al., 2025 (35)	2	Fisher’s Z = 1.29 [0.40, 2.18]	99	0.41
Perceived organizational support	Random-effects (DL)	Original (all studies)	—	3	Fisher’s Z = 0.65 [0.32, 0.97]	97	0.08
		Leave-one-out	Duan S. Y. et al., 2024 (30)	2	Fisher’s Z = 0.65 [0.05, 1.25]	98	0.18
		Leave-one-out	Jiang F. R. et al., 2023 (37)	2	Fisher’s Z = 0.80 [0.50, 1.10]	92	0.04
		Leave-one-out	Lian Q. et al., 2025 (35)	2	Fisher’s Z = 0.50 [0.19, 0.80]	95	0.05
Empowering leadership	Random-effects (DL)	Original (all studies)	—	2	Fisher’s Z = 0.67 [0.55, 0.79]	93	0.01
		Leave-one-out	Xu X. X. et al., 2023 (39)	1	—	—	—
		Leave-one-out	Wu M. R. et al., 2025 (28)	1	—	—	—

## 4 Discussion

This meta-analysis ultimately included 16 cross-sectional studies involving 11,678 Chinese clinical nurses. Pooled analyses showed that educational level, night shift frequency, employment type, self-efficacy, professional identity, perceived organizational support, and empowering leadership were factors associated with job crafting. Because the current evidence base is entirely limited to the Chinese healthcare system, the findings of this study primarily reflect the context of Chinese clinical nurses and should be extrapolated to other healthcare systems with caution.

Among these factors, the pooled results for self-efficacy, professional identity, perceived organizational support, and empowering leadership showed extremely high heterogeneity (I^2^ = 93 to 99%). This extremely high heterogeneity may mainly arise from differences among the included studies in measurement tools, sample characteristics, and geographic and organizational contexts. Therefore, the above pooled coefficients only reflect the average trend of effect sizes from existing studies under extremely high heterogeneity. These findings should be regarded as exploratory findings that may suggest possible associations rather than causal conclusions.

### 4.1 Occupational characteristics

The Job Demands-Resources (JD-R) model posits that job demands and job resources are two core job characteristics, which are associated with energy depletion and motivational processes, respectively (43). Employees are not passive recipients of job characteristics; rather, they proactively adjust their job demands and resources to maintain an optimal working state. Job crafting is a concrete manifestation of this proactive adjustment process, whereby employees redefine task, relational, and cognitive boundaries to promote the alignment between job demands and resources. When nurses have sufficient job resources and their job demands are within a tolerable range, they are more capable and more motivated to proactively reconstruct task, relational, and cognitive boundaries. Conversely, when resources are persistently depleted or scarce, proactive crafting behaviors may be inhibited.

Educational level. This meta-analysis showed that nurses with a junior college degree or below had lower job crafting scores than those with a bachelor’s degree or above (SMD = −0.30, 95% CI: −0.42 to −0.19), suggesting that educational level may be associated with nurses’ job crafting. The professional knowledge and critical thinking abilities acquired through higher education can be regarded as a personal resource (44), which may be related to nurses’ ability to mobilize opportunities in their work and implement job crafting strategies. A cross-sectional study of Iraqi nurses by Abdullah et al. (45) found that nurses with a master’s degree scored higher in knowledge, attitudes, and practice regarding evidence-based practice than those with a bachelor’s degree, and nurses with research experience had higher practice scores. This provides support for a positive association between educational level and proactive work behaviors among nurses.

Night shift frequency. This meta-analysis showed that nurses with a night shift frequency ≤6 times per month had higher job crafting scores than those with >6 times per month (SMD = 0.29, 95% CI: 0.18 to 0.40), suggesting that night shift frequency may be associated with nurses’ job crafting. Night shift frequency reflects the level of resource depletion associated with job demands. Previous studies have shown that frequent night shifts can deplete nurses’ physical and cognitive resources (46). When these resources are continuously depleted and difficult to recover, the resources available for proactive work adjustment may correspondingly decrease, which may be associated with lower levels of job crafting.

Employment type. This meta-analysis showed that permanent nurses had higher job crafting scores than non-permanent nurses (SMD = 0.56, 95% CI: 0.40 to 0.72), suggesting that employment type may be associated with nurses’ job crafting. This difference may be related to the dual employment system of permanent and non-permanent positions in Chinese public hospitals. Permanent nurses generally have more stable professional status, salary security, and career development pathways (47). In contrast, non-permanent nurses may face issues such as short-term contracts, lower remuneration, and limited promotion opportunities (48). These institutional resource differences may be related to nurses’ willingness and investment in proactively engaging in job crafting.

The above occupational characteristics suggest that nurses’ job crafting may be related to their disposable resources and institutional space. In talent management and scheduling system design, nursing managers may pay attention to the influence of these structural factors on nurses’ proactive behaviors. Supporting nurses’ continuing education and educational advancement, optimizing scheduling systems, and improving the welfare system for non-permanent nurses may help create more favorable conditions for job crafting. However, the above measures can be regarded as potential intervention targets, and their effectiveness still needs to be verified by prospective studies.

### 4.2 Psychological factors

Self-efficacy. This meta-analysis showed that self-efficacy was positively correlated with job crafting among nurses (pooled *r* = 0.60). Self-efficacy refers to an individual’s belief and confidence in their ability to complete a specific task or achieve an expected goal (49). According to social cognitive theory, self-efficacy influences individuals’ behavioral choices, effort, and persistence when facing challenges (50). Nurses with higher self-efficacy may be more likely to adopt adaptive coping strategies, such as active coping and planning, to address clinical challenges (51), which may be associated with higher levels of job crafting. However, this result was based on only 3 studies with extremely high heterogeneity (I^2^ = 99%); it should therefore be regarded as an exploratory association and interpreted with caution.

Professional identity. This meta-analysis showed that professional identity was positively correlated with job crafting among nurses (pooled *r* = 0.85). Professional identity refers to nurses’ positive cognition of the value, status, and responsibilities of the nursing profession, as well as the resulting positive behavioral tendencies (52). According to role identity theory, individuals tend to act in accordance with the expectations of the roles they identify with (53). Nurses with stronger professional identity may have a self-concept that is highly integrated with the nursing role, and may be more inclined to proactively adjust their work content and methods to meet their professional standards and role expectations. However, this result was based on only 3 studies with extremely high heterogeneity (I^2^ = 98%); the pooled coefficient may therefore be unstable and should be regarded as an exploratory association and interpreted with caution.

Both self-efficacy and professional identity are influenced by sociocultural contexts (54, 55). In the Chinese collectivist cultural context, the formation of self-efficacy may rely more on socially transmitted efficacy information, while professional identity may emphasize collective roles and responsibilities. Therefore, the pooled results for psychological factors based on Chinese nurses should be interpreted and extrapolated with caution in light of cultural context. The findings of this study suggest that training programs aimed at enhancing nurses’ self-efficacy or professional identity may theoretically help promote job crafting. However, the cross-sectional design cannot answer whether intervening on these psychological factors can truly improve job crafting. Future randomized controlled trials or longitudinal studies are needed to verify this.

### 4.3 Organizational factors

Perceived organizational support. This meta-analysis showed that perceived organizational support was positively correlated with job crafting among nurses (pooled r = 0.57). Perceived organizational support refers to an individual’s subjective perception of whether the organization provides help, care, and recognition (56). According to social exchange theory (57), social exchange refers to voluntary actions motivated by expected returns. Its core mechanism is the norm of reciprocity, which means that the recipient of help or resources is obliged to repay the giver. When nurses perceive organizational support, they may develop a sense of obligation to repay the organization based on the norm of reciprocity, and may respond to the organization by proactively adjusting their work tasks, relationships, and cognition. However, this result was based on only 3 studies with extremely high heterogeneity (I^2^ = 97%); the stability of the pooled estimate may therefore be limited.

Empowering leadership. This meta-analysis showed that empowering leadership was positively correlated with job crafting (pooled *r* = 0.58). Empowering leadership refers to a leadership behavior in which leaders, within the framework of organizational norms, delegate decision-making authority, provide autonomous working space, convey trust in subordinates’ abilities, and help them recognize the value of their work, thereby achieving power sharing (58). According to self-determination theory (59), autonomy, competence, and relatedness are three basic psychological needs, and their satisfaction is a key condition for maintaining intrinsic motivation. By granting nurses decision-making participation and autonomous space, and expressing trust in their abilities, empowering leadership may be related to the satisfaction of nurses’ autonomy and competence needs, and may be associated with enhanced intrinsic motivation for proactively engaging in job crafting. However, this result was based on only 2 studies with extremely high heterogeneity (I^2^ = 93%); this association still requires further verification.

There may be differences in the forms of organizational support and nursing management models across different healthcare systems. A study on structural empowerment in Chinese community nursing pointed out that Chinese nursing practice is constrained by hierarchical and resource-limited environments, with persistent powerlessness; in contrast, Western empowerment emphasizes professional autonomy and collective bargaining, and relies on unions and regulatory support to promote nurse-led models (60). Therefore, the results of this study are based on the Chinese nursing context, and this institutional difference should be considered when extrapolating. The results of this meta-analysis suggest that nursing managers may pay attention to the development of organizational support systems and empowering leadership behaviors. Improving resource assurance, granting nurses more autonomous decision-making space, and building a supportive and empowering organizational culture may help create a more favorable organizational environment for job crafting. However, these measures are currently proposed only based on cross-sectional associations, and their effectiveness still needs to be verified by future research.

### 4.4 Limitations

This study has several limitations. First, all included studies were cross-sectional, precluding the establishment of causal relationships. Second, all included studies were conducted in China, and the evidence base is entirely limited to the Chinese healthcare system; therefore, the applicability of the conclusions to different medical and cultural contexts requires further validation. Third, each meta-analysis included only 2–4 studies, which limits the precision of the pooled estimates and restricts the adequate exploration of sources of heterogeneity and publication bias. Fourth, some factors exhibited extremely high heterogeneity (I^2^ > 90%), and the certainty of evidence assessed by GRADE was rated as very low, limiting the reliability of the pooled results. Given these limitations, the conclusions of this study should be regarded as exploratory findings and should be interpreted and generalized with caution.

## 5 Conclusion

Identifying factors associated with job crafting among nurses can help nursing managers focus on conditions that may influence nurses’ proactive adjustments to their work. This study synthesized data from 16 cross-sectional studies involving 11,678 Chinese clinical nurses through a systematic review and meta-analysis. Educational level, night shift frequency, employment type, self-efficacy, professional identity, perceived organizational support, and empowering leadership were preliminarily identified as factors associated with job crafting. Because all included studies were cross-sectional studies of Chinese clinical nurses, some factors had high heterogeneity and the number of included studies was limited, the pooled results are subject to considerable uncertainty. Therefore, these findings should be considered exploratory evidence and may inform future longitudinal and intervention studies. Future cross-cultural, longitudinal, or intervention studies are needed to verify the causal relationships and intervention effectiveness of these associated factors, so as to provide higher-quality evidence for developing targeted nursing management strategies.

## Data Availability

The original contributions presented in the study are included in the article/Supplementary material, further inquiries can be directed to the corresponding author.
