# Supplementary material for: Factors associated with job crafting among Chinese clinical nurses-a systematic review and meta-analysis
Source: Front Public Health. 2026 Oct 19;14:1961368. doi: 10.3389/fpubh.2026.1961368 (PMC13724421; doi:10.3389/fpubh.2026.1961368)
Supplement: Supplementary file 2 [file Table_2.DOCX]

**Appendix A: Search strategy**

| **Database** | **Search terms** | **Results** |
| --- | --- | --- |
| PubMed | #1 "Nurses"[Mesh] OR "nurse"[Title/Abstract] OR "nursing personnel"[Title/Abstract] OR "personnel, nursing"[Title/Abstract] OR "registered nurses"[Title/Abstract] OR "nurse, registered"[Title/Abstract] OR "nurses, registered"[Title/Abstract] OR "registered nurse"[Title/Abstract] OR "nurs*"[Title/Abstract]  #2 "job crafting"[Title/Abstract] OR "job design"[Title/Abstract] OR "job adjustment"[Title/Abstract] OR "job changes"[Title/Abstract] OR "crafting"[Title/Abstract] OR "task crafting"[Title/Abstract] OR "cognitive crafting"[Title/Abstract] OR "relational crafting"[Title/Abstract] OR "seeking resources"[Title/Abstract] OR "seeking challenges"[Title/Abstract] OR "reducing hinder demands"[Title/Abstract] OR "increasing structural job resources"[Title/Abstract] OR "increasing social job resources"[Title/Abstract] OR "decreasing hindering job demands"[Title/Abstract] OR "increasing challenging job demands"[Title/Abstract]  #3 #1 AND #2 | 126 |
| Cochrane Library | #1(nurse):ab,ti,kw OR (nursing personnel):ab,ti,kw OR (personnel nursing):ab,ti,kw OR (registered nurses):ab,ti,kw OR (nurse registered):ab,ti,kw OR (nurses registered):ab,ti,kw OR (registered nurse):ab,ti,kw OR (nurs*):ab,ti,kw  #2 (job crafting):ab,ti,kw OR (job design):ab,ti,kw OR (job adjustment):ab,ti,kw OR (job changes):ab,ti,kw OR (crafting):ab,ti,kw OR (task crafting):ab,ti,kw OR (cognitive crafting):ab,ti,kw OR (relational crafting):ab,ti,kw OR (seeking resources):ab,ti,kw OR (seeking challenges):ab,ti,kw OR (reducing hinder demands):ab,ti,kw OR (increasing structural job resources):ab,ti,kw OR (increasing social job resources):ab,ti,kw OR (decreasing hindering job demands):ab,ti,kw OR (increasing challenging job demands):ab,ti,kw  #3 #1 AND #2 | 473 |
| Embase | #1 'nurses':ab,ti OR 'nursing personnel':ab,ti OR 'personnel nursing':ab,ti OR 'registered nurses':ab,ti OR 'nurse registered':ab,ti OR 'nurses registered':ab,ti OR 'registered nurse':ab,ti OR 'nurs*':ab,ti  #2 'job crafting':ab,ti OR 'job design':ab,ti OR 'job adjustment':ab,ti OR 'job changes':ab,ti OR 'crafting':ab,ti OR 'task crafting':ab,ti OR 'cognitive crafting':ab,ti OR 'relational crafting':ab,ti OR 'seeking resources':ab,ti OR 'seeking challenges':ab,ti OR 'reducing hinder demands':ab,ti OR 'increasing structural job resources':ab,ti OR 'increasing social job resources':ab,ti OR 'decreasing hindering job demands':ab,ti OR 'increasing challenging job demands':ab,ti  #3 #1 AND #2 | 241 |
| Web of Science | #1 TS=("nurses" OR "nurse" OR "nursing personnel" OR "personnel nursing" OR "registered nurses" OR "nurse registered" OR "nurses registered" OR "registered nurse" OR "nurs*")  #2 TS=("job crafting" OR "job design" OR "job adjustment" OR "job changes" OR "crafting" OR "task crafting" OR "cognitive crafting" OR "relational crafting" OR "seeking resources" OR "seeking challenges" OR "reducing hinder demands" OR "increasing structural job resources" OR "increasing social job resources" OR "decreasing hindering job demands" OR "increasing challenging job demands")  #3 #1 AND #2 | 155 |
| Scopus | #1 TITLE-ABS-KEY("nurses" OR "nurse" OR "nursing personnel" OR "personnel, nursing" OR "registered nurses" OR "nurse, registered" OR "nurses, registered" OR "registered nurse" OR "nurs*")  #2 TITLE-ABS-KEY("job crafting" OR "job design" OR "job adjustment" OR "job changes" OR "crafting" OR "task crafting" OR "cognitive crafting" OR "relational crafting" OR "seeking resources" OR "seeking challenges" OR "reducing hinder demands" OR "increasing structural job resources" OR "increasing social job resources" OR "decreasing hindering job demands" OR "increasing challenging job demands")  #3 #1 AND #2 | 378 |
| CNKI | #1 SU=(护士OR临床护士OR护理人员OR注册护士)  #2 SU=(工作重塑OR工作形塑)  #3 #1 AND #2 | 94 |
| Wanfang | #1 (主题:("护士") OR 主题:("临床护士") OR 主题:("护理人员") OR 主题:("注册护士"))  #2 (主题:("工作重塑") OR 主题:("工作形塑"))  #3 #1 AND #2 | 474 |
| VIP | #1 M=(护士 OR 临床护士 OR 护理人员 OR 注册护士)  #2 M=(工作重塑 OR 工作形塑)  #3 #1 AND #2 | 56 |
| CBM | #1 ("护士"[常用字段:智能] OR "临床护士"[常用字段:智能] OR "注册护士"[常用字段:智能] OR "护理人员"[常用字段:智能])  #2 ("工作重塑"[常用字段:智能] OR "工作形塑"[常用字段:智能])  #3 #1 AND #2 | 73 |
